# Grandchild Care and Grandparents’ Well-Being in Context: The Impact of the COVID-19 pandemic

**DOI:** 10.1093/geroni/igae101

**Published:** 2024-11-02

**Authors:** Mareike Bünning, Oliver Huxhold

**Affiliations:** German Centre of Gerontology, Berlin, Germany; German Centre of Gerontology, Berlin, Germany

**Keywords:** COVID-19, Gender Issues, Life Satisfaction, Loneliness, Quantitative research methods

## Abstract

**Background and Objectives:**

This study investigates whether the association between supplementary grandchild care and grandparents’ subjective well-being—measured as life satisfaction, perceived stress, and loneliness—is moderated by the contextual environment. We use the coronavirus disease (COVID-19) pandemic as an example of contextual differences. Drawing on role theory, we argue that the costs and benefits of grandparenting may have differed between pandemic and prepandemic times. On the one hand, providing grandchild care during the pandemic may have been particularly stressful, prompting more negative effects on well-being. On the other hand, grandchild care may have been particularly relevant for enhancing well-being, as it protected grandparents from social isolation. Moreover, the association between grandparenting and well-being may have differed by gender.

**Research Design and Methods:**

Using unbalanced panel data from the German Ageing Survey (DEAS) 2014 (*n* = 3,619), 2017 (*n* = 2,458), and 2020 (*n* = 2,021), we applied maximum likelihood structural equation modeling (ML-SEM)**—**a method that combines dynamic panel modeling with fixed-effects analysis**—**to examine whether there were differences in the relationship between grandchild care and grandparents’ well-being when comparing pandemic and prepandemic times and by grandparents’ gender.

**Results:**

Grandchild care was associated with lower loneliness for both grandmothers and grandfathers. For grandfathers, this association was even stronger during the pandemic. Grandmothers experienced higher life satisfaction when taking care of grandchildren during the pandemic, but there was no evidence that grandchild care increased perceived stress for either grandmothers or grandfathers.

**Discussion and Implications:**

In line with role enhancement theory, this study highlights that supplementary grandchild care can be beneficial for grandparents’ well-being. Moreover, the context in which grandchild care takes place shapes the costs and rewards associated with it. Our results suggest that supportive policies and programs facilitating grandchild care can enhance grandparents’ well-being, especially in challenging contexts.

**Translational Significance:** This study examined how caring for a grandchild influenced the well-being of grandfathers and grandmothers before and during coronavirus disease (COVID-19) pandemic. The results showed that supplementary grandchild care reduced loneliness for both genders, especially during the pandemic. Grandmothers, but not grandfathers, experienced increased life satisfaction during the pandemic. Additionally, supplementary grandchild care was unrelated to stress in both genders at all times. Overall, our findings provide further evidence that well-being gains depend on the situational and societal context, in particular the opportunity structure for social interactions. To this end, targeted, low-level initiatives addressing grandmothers and grandfathers alike, may be effective.

## Background and Objectives

Many grandparents around the globe regularly take care of grandchildren who live outside their household (e.g., Hank & Buber, 2009, on Europe; Ko & Hank, 2014, on Asia; Luo et al., 2012, on the United States). This supplementary grandchild care allows them to establish a close bond with their grandchildren (Brown, 2003), foster intergenerational solidarity (Bengtson & Roberts, 1991), and support maternal employment—especially when access to daycare is limited (Arpino et al., 2014; Bünning, 2017). A great number of studies have explored how supplementary grandchild care is related to grandparents’ subjective well-being. These studies, which considered a broad range of indicators for well-being and diverse cultural contexts, have found predominantly positive associations when using cross-sectional data; results were more mixed in studies with longitudinal designs (see e.g., reviews by Danielsbacka et al., 2022; Kim et al., 2017, for an overview).

In this study, we assume that the societal and situational context in which grandparenting takes place moderates the association between grandchild care and grandparents’ well-being. In particular, drawing on role enhancement theory and role strain theory, we argue that the coronavirus disease (COVID-19) pandemic may have had a profound impact on the relationship between grandchild care and grandparents’ well-being. There may have been different costs and benefits of engaging in caring grandparenting during the pandemic than before. On the one hand, grandchild care during the pandemic might have been particularly stressful, meaning it had a more negative effect on well-being than before the pandemic. On the other hand, grandchild care may have been particularly relevant for enhancing well-being during the pandemic, as it may have protected grandparents from social isolation.

This study hence contributes to the literature by examining how the COVID-19 pandemic altered the association between grandchild care and three aspects of subjective well-being: life satisfaction (Diener et al., 1985), perceived stress (Cohen et al., 1983), and loneliness (De Jong Gierveld & van Tilburg, 2006). Life satisfaction can be understood as the global assessment of an individual’s subjective quality of life. The pandemic might have particularly affected perceived stress and loneliness because there was a perceived threat to the individual’s health and because enforced social distancing had unintended consequences (Gilligan et al., 2020). Moreover, given that men and women perform different social roles, we compared the situation of grandmothers to that of grandfathers.

### Grandchild Care in Germany Before and During the COVID-19 Pandemic

In Germany, about one third of grandparents regularly take care of their grandchildren. In the European context, Germany holds an intermediary position, falling between the Nordic countries, which have high rates of low-intensity grandchild care, and the southern European countries, where grandparental involvement is less common but more intensive when it occurs (Hank & Buber, 2009; Zanasi et al., 2023). This pattern can be attributed to varying family policies across Europe. In Nordic countries, high levels of defamilization mean that publicly funded services relieve families of many caregiving duties. In contrast, Germany, like many central European countries, follows a model of supported familialism, where financial support and the availability of part-time jobs enable families to maintain their caregiving roles. Meanwhile, in southern and central-eastern European countries, a model of “familism by default” prevails, where family members are often compelled to provide care due to the scarcity of publicly available alternatives (Saraceno & Keck, 2010; Zanasi et al., 2023). Before the pandemic, grandmothers were involved in grandchild care more often than grandfathers. Grandchild care was more common among grandparents aged 60–69 years than among both older and younger grandparents, whereas there was no difference by educational background. Moreover, geographical proximity was decisive in whether grandparents took care of their grandchildren: Grandparents who lived in the same city as their grandchildren were twice as likely to engage in grandchild care as those who lived further away (Bünning et al., 2023).

During the COVID-19 pandemic, measures were implemented in Germany to slow down the spread of the virus. The data for the present analysis were collected in winter 2020/2021 (November to March) when the second wave of the pandemic hit Germany. Social distancing measures and limits on social contact were in place from November 2020 onward and were tightened in mid-December, when schools and daycare centers were closed for several weeks. The 7-day incidence rate peaked at 197 per 100 000 inhabitants (RKI, 2023), and the number of COVID-19 deaths peaked at 6,000 per week at the turn of the year 2020/21 (RKI, 2024). The first vaccinations against COVID-19 became available in Germany at the end of 2020, so the vast majority of grandparents had not yet been vaccinated at the time of data collection.

Nevertheless, the prevalence and composition of grandparents who provided grandchild care remained remarkably stable. As a recent descriptive analysis from Germany has shown, neither the proportion of grandparents providing grandchild care nor the average number of childcare hours provided by grandparents changed significantly between 2017 and the winter of 2020/2021 (Bünning et al., 2023). This trend did not differ between grandmothers and grandfathers or between educational groups. Regarding age differences, there was a slight decline in grandchild care provided by grandparents aged 60–69, but there were no changes in grandchild care behavior among older and younger grandparents. Even among grandparents with pre-existing health conditions—such as chronic lung diseases, cardiovascular diseases, or cancer—the proportion of grandparents who provided grandchild care remained remarkably stable; only grandparents with overweight slightly decreased their grandchild care provision. Moreover, grandparents who lived further away from their grandchildren provided grandchild care less often in winter 2020/2021 than in 2017; for those living nearby, childcare rates remained stable.

### Role Strain and Role Enhancement Among Caregiving Grandparents

We employed role theory to analyze the relationship between grandparental child care and well-being before and during the COVID-19 pandemic. Specifically, we drew on two contrasting perspectives, role enhancement theory and role strain theory, to highlight the benefits and risks associated with performing an active role as a grandparent who provides grandchild care.

According to role enhancement theory, there are many beneficial aspects of social roles such as resources, status, fulfillment, or personal growth. Thus, performing different social roles, the grandparental role among them, can be beneficial for well-being (Moen et al., 1995; Rozario et al., 2004; Sieber, 1974). For instance, providing grandchild care may embed grandparents in intergenerational family networks and may hence protect them from social isolation (Zhang et al., 2021).

A contrasting perspective is offered by role strain theory, which posits that people have limited time and energy to perform multiple roles. Accordingly, attempts to perform too many roles may lead to role conflicts, stress, and overload (Goode, 1960; Moen et al., 1995). Moreover, role strain may arise when specific behaviors required by one role conflict expectations placed on another role (Greenhaus & Beutell, 1985). Grandchild care may hence limit the time and energy available for other activities that might foster grandparents’ well-being or compromise well-being because of behavior-based conflicts with other social roles.

Studies conducted before the pandemic have generated inconclusive results on whether grandparents providing supplementary grandchild care are more likely to experience positive or negative well-being consequences, that is, whether role enhancement or role strain dominates. Studies using cross-sectional designs have tended to find positive associations between supplementary grandchild care provision and well-being (see review by Danielsbacka et al., 2022). Yet, these results do not necessarily confirm that active grandparenting has beneficial effects. Instead, selection processes or reverse causality may explain these positive associations. In other words, more healthy grandparents and those with elevated levels of well-being could be more likely to take care of their grandchildren than less healthy grandparents with lower levels of well-being.

Studies using longitudinal designs or instrumental variable regression to tackle these issues have yielded ambiguous results. Whereas some studies have found positive effects of grandchild care on well-being (Di Gessa et al., 2016; Hughes et al., 2007; Ku et al., 2012; Notter, 2022; Zhang et al., 2021), others have been unable to establish significant associations (Bordone & Arpino, 2019; Danielsbacka et al., 2019; Ku et al., 2013) or have even found negative effects (Komonpaisarn & Loichinger, 2019; Ku et al., 2012). Qualitative research has also provided evidence of both increases and decreases in well-being due to grandchild care (Derrer-Merk et al. 2024).

These ambiguous results could imply that grandchild care has a less pronounced effect on grandparents’ well-being than theoretically assumed. However, the ambiguous empirical findings could also indicate the existence of influential moderator variables (Hedges & Pigott, 2004). Support for this intuition is provided by previous studies, which have found that the societal and situational context of grandparenting plays an important role in the relationship between grandchild care and well-being. For example, studies have repeatedly found more negative well-being outcomes for custodial grandparents or grandparents who live in the same household as their grandchildren than for grandparents who provide supplementary care for grandchildren with whom they do not reside (see review by Danielsbacka et al., 2022). In addition, Bordone and Arpino (2019) have found considerable cross-national differences in the relationship between grandchild care and well-being across 15 European countries and Israel, though country patterns did not align with the family policy regimes identified by Saraceno and Keck (2010): Grandchild care was unrelated to depressive symptoms among grandmothers in Germany, whereas grandmothers in France showed a reduction in depressive symptoms when caring for their grandchildren, wheras grandmothers in Poland and Slovenia showed increased depressive symptoms when caring for their grandchildren. Intensive grandchild care, moreover, was related to increased depressive symptoms among grandmothers in Israel, Spain, Estonia, and Sweden. Regarding grandfathers, intensive grandchild care was associated with reductions in depressive symptoms in Germany and Belgium, but increases in depressive symptoms in Italy and the Czech Republic. Finally, Neuberger and Haberkern (2014) found some evidence that normative societal expectations about grandparenting moderated associations between grandchild care and grandparents’ quality of life.

### The COVID-19 Pandemic as a Moderating Context

In this study, we used the emergence of the COVID-19 pandemic to examine how the contextual situation shapes the effect of grandchild care on grandparent’s well-being. The COVID-19 pandemic dramatically changed the societal and situational context in which grandchild care was embedded. To slow down the spread of the virus, all people, especially older adults, were called on to self-isolate. At the same time, schools and daycare centers were closed, putting families under pressure to cope with additional childcare and homeschooling demands. The pandemic hence created a “connectivity paradox” (Smith et al., 2020) as people needed to stay apart at a time when there was an increased need for instrumental and emotional support.

The ambivalence that this situation provoked may have had an impact on both role enhancement and role strain associated with grandchild care. From the perspective of role enhancement, grandchild care may have been particularly relevant in protecting grandparents from social isolation during the pandemic, when social distancing measures required older people to self-isolate. Reduced physical contact, though effective in reducing the spread of the virus, may have had detrimental effects on individuals’ mental health and well-being; indeed, these effects have been discussed as one of the most severe unintended consequences of social distancing measures (Chiesa et al., 2021).

Yet, role strain may also have increased during the pandemic. One reason for this is that the role of a grandparent who wanted to support their grandchildren and adult children during a time of crisis conflicted with the role of the good citizen who self-isolated to prevent the spread of the virus. Prepandemic studies found that violating normative social expectations was stressful and associated with reduced well-being (Neuberger & Haberkern, 2014). Moreover, grandparents may have perceived grandchild care as more stressful during the pandemic than in prepandemic times because they were afraid to contract the virus from their children and grandchildren. Grandchild care may also have been more challenging because grandchildren may have been more distressed due to the consequences of the pandemic (Gilligan et al., 2020), and the restrictions on formal daycare provision might have required relatives to spend more time providing private childcare. Moreover, social distancing requirements that called on people to limit contact to a set “pod” of people may have prompted grandparents who provided childcare to sacrifice other social contacts and activities.

To our knowledge, only one quantitative study so far has covered the relationship between grandparenting and well-being during the COVID-19 pandemic (Di Gessa et al., 2022). Using data from England, this study found that grandparents who stopped taking care of their grandchildren or reduced the amount of grandchild care during the pandemic reported poorer mental health than those who maintained prepandemic levels of grandchild care. This is the first evidence that role enhancement was more pronounced than role strain with regard to grandchild care during the pandemic (see also Derrer-Merk et al. 2024, for qualitative research with similar findings). Yet, the study could not say whether this association was specific to the pandemic context, as it did not investigate whether stopping grandchild care led to similar declines in mental health before the pandemic.

### Gender Differences in the Relationship Between Grandchild Care and Well-Being

The association between grandchild care and well-being in the pandemic may also have differed by gender. In general, interpersonal relationships tend to be more important for women’s well-being than for men’s (Joshanloo, 2018). Women often take on the role of kin-keeper or family caregiver (Rosenthal, 1985). Whereas grandmothers often view grandchild care as a right and expectation, grandfathers may see it as voluntary (Stelle et al., 2010). Consequently, grandmothers are usually more involved in grandchild care than grandfathers, they spend more time with their grandchildren without their spouse being present, and they often take on the more demanding routine child-care tasks, wheras grandfathers primarily play with their grandchildren (Craig & Jenkins, 2015; Winefield & Air, 2010). Moreover, grandmothers may perceive taking care of grandchildren as a continuation of their role as mothers.

Consistent with these differences in social roles, some longitudinal studies indicate that supplementary grandchild care improves grandmothers’ well-being more than grandfathers’ (Hughes et al., 2007; Notter, 2022). However, other studies have reported more ambiguous and context-dependent results. For example, Grundy et al. (2012) found that grandchild care was related to greater life satisfaction among grandfathers but not grandmothers, whereas grandmothers who took care of their grandchildren had lower risks of reporting depression symptoms, but grandfathers did not. Moreover, during the pandemic, strain may have increased for men to a greater extent than for women, as men had a higher risk of becoming severely ill with COVID-19 (Gao et al., 2021).

### Hypotheses

Based on the theoretical arguments and the study findings outlined earlier, we derived the following hypotheses regarding the effect of grandchild care on grandparents’ well-being during the pandemic. Drawing on role strain theory, we expected grandchild care to be more stressful during the pandemic. We hence hypothesized that grandparents who took care of their grandchildren during the COVID-19 pandemic experienced elevated levels of stress (H1). In contrast, drawing on role enhancement theory, we expected grandchild care to have been more important for social integration, as contact restrictions reduced available alternatives. We hence hypothesized that grandparents taking care of their grandchildren during the COVID-19 pandemic would feel less lonely (H2) and more satisfied with their lives (H3). Moreover, we expected grandchild care to enhance well-being more among grandmothers than grandfathers (H4). In essence, we expect grandmothers to experience less stress, less loneliness, and greater life satisfaction compared to grandfathers when caring for their grandchildren.

## Research Design and Methods

### Data

We drew on data from the German Ageing Survey (DEAS, https://doi.org/10.5156/DEAS.1996-2021.M.002; Vogel et al., 2021). DEAS is a representative cohort-sequential study. Baseline samples (collected in 1996, 2002, 2008, and 2014) were drawn from a random sample of municipalities and stratified by age, gender, and region (Western and Eastern Germany). Respondents were then contacted again for interviews every 3 years unless they withdrew panel consent or dropped out of the sample due to death, permanent illness, or a move abroad. For this study, we used the three most recent waves of DEAS (2014, 2017, and 2020), which included participants from all baseline samples. The data hence cover two prepandemic periods as well as one measurement occasion during the COVID-19 pandemic. Data collection consisted of a personal interview and a self-administered questionnaire that was filled out by about 85% of respondents (Vogel et al., 2021). Information on grandchildren and grandchild care was collected in the personal interview; information on subjective well-being was gathered in the self-administered questionnaire.

We restricted the sample to respondents who had at least one grandchild below age 14. Grandparents who only had older grandchildren were excluded because children aged 14 and above were likely able to cope without supervision when schools were closed and parents had to work (Zoch et al., 2021). Moreover, grandparents who lived together with a grandchild in a joint household for at least 1 year were excluded because of small case numbers. Coresiding grandparents were presumably much more involved in caregiving than non-coresiding grandparents, and thus, the two groups may have had different experiences and consequences for well-being (Danielsbacka et al., 2022; note that unlike in the United States, custodial grandparenthood is very uncommon in Germany. In our sample, only 39 grandparents lived in a household with a grandchild but without their child [in-law] in 2014. The respective numbers for 2017 and 2020 were 18 and 14). Our design left us with an unbalanced panel with *n* = 3,619 in 2014, *n* = 2,458 in 2017, and *n* = 2,021 in 2020. An overview of the cases excluded in each step is provided in Table 1. Altogether, 27% of respondents participated in all three waves, 27% participated twice, and 46% participated only once.

**Table 1.** Sample Selection. Case Numbers Left After Each Exclusion Step by Wave of Data Collection

	2014	2017	2020
Full sample	10,323	6,626	5,402
Had grandchild(ren) below age 14	3,743	2,538	2,076
No grandchild living in household	3,619	2,458	2,021

### Variables

We used three indicators to measure subjective well-being: life satisfaction, loneliness, and stress. Life satisfaction refers to an overall cognitive judgment of one’s quality of life according to one’s chosen criteria (Diener et al., 1985). We used the Satisfaction with Life Scale developed by Diener et al. (1985). It consists of five items that are measured on a five-point scale, ranging from 1 (*strongly disagree*) to 5 (*strongly agree*). Example items included “In most ways my life is close to my ideal” and “The conditions of my life are excellent.” Higher values indicated greater life satisfaction.

Loneliness arises when the number of relationships one has is fewer than what is desired, or when the level of intimacy one seeks has not been realized. We assessed loneliness with the six-item scale by De Jong Gierveld and van Tilburg (2006). Items were measured on a four-point scale ranging from 1 (*strongly agree*) to 4 (*strongly disagree*). Example items included “I often feel rejected” and “There are enough people I feel close to.” Item scores were recoded so that higher values indicated greater loneliness.

Perceived stress results from encountering a threatening or demanding situation and lacking the resources to cope with it (Cohen et al., 1983). We used the four-item perceived stress scale developed by Cohen et al. (1983). Items were measured on a 5-point scale ranging from 1 (*never*) to 5 (*very often*). Example items were “In the last month, how often have you felt difficulties were piling up so high that you could not overcome them?” and “In the last month, how often have you felt that you were unable to control the important things in your life?” Higher values indicated more elevated stress.

Our main independent variable was grandchild care. Respondents were asked “Do you look after or supervise other people’s children privately?” and presented with the following options: (a) grandchildren; (b) children of siblings; (c) children of neighbors; (d) children of friends or acquaintances; (e) other; or (f) no. We operationalized grandchild care as a dummy variable that took the value of 1 if respondents answered “yes” to the “grandchildren” category and 0 otherwise.

In addition, we controlled for the following time-varying variables: self-rated health (measured on a 5-point scale ranging from 1 (*very bad*) to 5 (*very good*)), partnership status (1 if the respondent lived with a partner in the same household, 0 otherwise), and employment status (1 employed, 0 not employed, retired). Moreover, the models included two time-constant variables: year of birth (linear, divided by 10) and level of education (low, medium, high according to the International Standard Classification of Education [ISCED]). All control variables were centered around their grand mean in 2017 and their effects were allowed to vary between men and women. Descriptive statistics on all dependent and independent variables are provided in Supplementary Table 1.

### Method

Given that selection into grandparenting explains a considerable portion of the association between grandchild care and well-being and that the composition of grandparents providing grandchild care was slightly different during the pandemic than in pre-pandemic times, we thought it important to factor out selection effects to study the effect of grandchild care on grandparents’ well-being before and during the pandemic. We, therefore, used the maximum-likelihood structural equation modeling (ML-SEM) approach developed by Allison et al. (2017) and Moral-Benito et al. (2019). This approach combines a dynamic panel model with fixed-effects analysis. Using fixed effects allowed us to net out unobserved time-invariant confounders and thus addressed selection based on unobserved time-constant characteristics. We included observed time-varying covariates in the model to control for time-varying confounders. In addition, we used a dynamic panel model to separate the effect of grandchild care on well-being from the reverse causal pathway, which implies that grandparents’ well-being may influence their decision to care for their grandchildren. Previous research has either used fixed effects (Bordone & Arpino, 2019; Danielsbacka et al., 2019; Ku et al., 2012; Notter, 2022) or lagged dependent variables (Di Gessa et al., 2016, 2022; Hughes et al., 2007; Ku et al., 2013; Zhang et al., 2021), but, to our knowledge, no study to date has combined dynamic panel models with fixed effects to exploit the strengths of both approaches.

**Figure 1.**
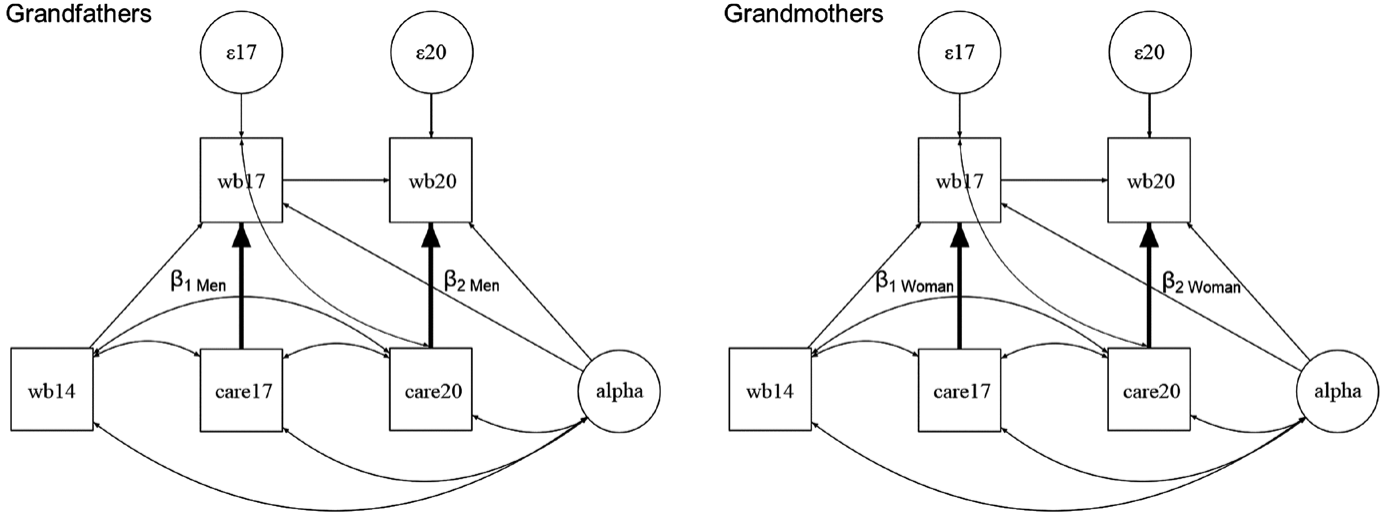
Analytical model. wb = well-being (separate models for loneliness, perceived stress, life satisfaction), care = grandchild care, 14 = year 2014, 17 = year 2017, 20 = year 2020, β = main effect of interest, ε = error term.

The path diagram of the model we estimated is displayed in Figure 1. We estimated a multigroup model to test separate structural models for men and women. We conducted separate models for each dependent variable. We were mainly interested in assessing the effect of grandchild care (care) on our measures of well-being (wb). We wanted to test whether these relationships differed before the pandemic (2017) and during it (2020) as well as between genders. To do so, we separately estimated the coefficients β_1_ (effect of grandchild care on well-being in 2017) and β_2_ (effect of grandchild care on well-being in 2020) for men and women. We then used Chi² tests to assess whether the effect of grandchild care on well-being differed between 2017 and 2020 (β_1Men_ vs β_2Men_ and β_1Women_ vs β_2Women_), and between grandfathers and grandmothers (β_1Men_ vs β_1Women_ and β_2Men_ vs β_2Women_).

In addition to the variables displayed in Figure 1, the model included self-rated health, partnership status, and employment status as time-varying covariates, as well as year of birth and level of education as time-invariant variables. All control variables were allowed to vary between men and women.

To control for unobserved time-invariant confounders, we designed the model to include the latent variable α, which had unrestricted correlations with all time-varying predictors (lagged well-being, current grandchild care, and time-varying control variables). The time constant control variables were uncorrelated with α. We hence obtained equivalent results by estimating α as when using fixed-effects panel models.

Moreover, we used a dynamic panel model to model the direction of the causal relationships. This included two components. The first was a lagged effect of the dependent variable on itself; that is, we used well-being in 2014 [2017] as an independent variable to estimate well-being in 2017 [2020]. Second, we modeled grandchild care as predetermined rather than strictly exogenous; that is, we allowed the error term of well-being to correlate with future values of grandchild care and thus accommodated feedback effects from well-being on grandchild care. In line with standard econometrics procedures and in contrast to psychological approaches, we did not lag the independent variables because we thought that a 3-year time lag would be too long to expect a meaningful relationship between lagged grandchild care behavior and current well-being and because we were interested in whether the pandemic had changed the relationship between current grandchild care behavior and current well-being.

One potential problem that we were aware of from previous research was that the combination of fixed-effects models with dynamic panel models could result in serious estimation problems because the lagged dependent variable would not be independent of the person-specific error term u_i_, thus violating a pivotal assumption of fixed-effects models. Yet, we solved this problem by specifying that grandchild care was predetermined rather than strictly exogenous. As demonstrated by previous research (Allison et al., 2017; Moral-Benito et al., 2019), this approach is less biased and more efficient than approaches using the generalized method of moments to overcome estimation bias.

We used MPlus (Version 8.4, Muthén & Muthén, 2017) to estimate the multigroup structural equation model outlined earlier. We used full-information maximum likelihood to handle missing values and longitudinal attrition. In the following section, we present the results from the model that yielded the best fit for each dependent variable based on the chi-square tests outlined above. Root mean square error of approximation (RMSEA) and comparative fit index (CFI) values indicated that each of these models had a good fit.

## Results

Results from the multigroup maximum-likelihood structural equation models are displayed in Table 2. As shown in Column 1, we found that grandchild care was unrelated to perceived stress among both grandfathers and grandmothers, in 2017 and 2020 alike. We hence found no support for Hypothesis 1 that grandparents taking care of their grandchildren during the COVID-19 pandemic experienced elevated levels of stress.

**Table 2.** β-Coefficients and Standard Errors From Multigroup Maximum Likelihood Structural Equation Models

	Stress^a^		Loneliness^b^		Life satisfaction^c^	
Grandfathers	β	*SE*	β	*SE*	β	*SE*
Child care 2017						
β_1Men_	0.02	0.05	-0.10^*^	0.04	0.08	0.05
Child care 2020						
β_2Men_	0.02	0.05	-0.26^**^	0.09	0.08	0.05
Grandmothers						
Child care 2017						
β_1Women_	0.02	0.05	-0.10^*^	0.04	0.08	0.05
Child care 2020						
β_2Women_	0.02	0.05	-0.26^**^	0.09	0.35^***^	0.09
RMSEA	0.024		0.005		0.025	
CFI	0.994		1.000		0.995	

*Notes:* Stress: five-point scale of four items, higher values indicated more elevated stress. Loneliness: four-point scale of six items, higher values indicated greater loneliness. Life satisfaction: five-point scale of five items, higher values indicated greater life satisfaction. Child care: dummy variable (reference category: no child care). The models controlled for lagged values of the dependent variable, self-rated health, partnership status, employment status, year of birth, level of education. Full estimation results are available in Supplementary Table 2. CFI = comparative fit index; RMSEA = root mean square error of approximation; *SE* = standard error.

^a^All coefficients (β_1Men_, β_1Women_, β_2Men_, and β_2Women_) set equal.

^b^β_1Men_ and β_1Women_ set equal, β_2Men_, and β_2Women_ set equal.

^c^β_1Men_, β_2Men_, and β_1Women_ set equal.

^*^ *p < *.05.

^**^ *p* < .01.

^***^ *p* < .001.

Column 2 shows that grandchild care was negatively associated with loneliness for grandmothers and grandfathers, both in 2017 and 2020. Before the pandemic, grandparents taking care of grandchildren felt 0.1 scale points less lonely, on a four-point scale, than grandparents who did not take care of their grandchildren. During the pandemic, this association more than double to 0.26 scale points. Moreover, among grandfathers, chi-square tests indicated that the effect of grandchild care on loneliness was stronger in 2020 than in 2017. Among grandmothers, by contrast, chi-square tests indicated no difference in the association between grandchild care and loneliness between the two time periods. Gender differences in the effect of grandchild care on loneliness did not differ significantly, either in 2017 or 2020. The results were in line with Hypothesis 2 that grandchild care would protect grandparents against loneliness, particularly during the COVID-19 pandemic.

Column 3 shows that grandchild care was unrelated to grandfathers’ life satisfaction both in 2017 and 2020. Likewise, grandchild care was unrelated to grandmothers’ life satisfaction in 2017. Yet, in 2020, we found a strong, positive effect of grandchild care on life satisfaction among grandmothers. Grandmothers taking care of grandchildren were 0.35 scale points more satisfied with their lives, on a 5-point scale than grandmothers who did not take care of their grandchildren. A chi-square test indeed showed that the effect of grandchild care on life satisfaction among grandmothers was stronger in 2020 than in 2017. Moreover, whereas chi-square tests did not detect significant gender differences in the relation between grandchild care and life satisfaction in 2017, grandchild care was more important for life satisfaction among grandmothers than grandfathers in 2020. The results hence partially support Hypothesis 3 that grandparents taking care of their grandchildren during the COVID-19 pandemic were more satisfied with their lives (H3). Moreover, they are in line with Hypothesis 4, which postulated that grandchild care would enhance the well-being of grandmothers to a greater extent than the well-being of grandfathers (H4). To ensure the robustness of our findings, we conducted additional analyses to check whether the results remained consistent under different conditions. Specifically, we (a) included grandparents living with their grandchildren in the sample, and (b) excluded those who provide intensive childcare (defined as at least 15 hr/wk, following Di Gessa et al. [2016]). The results remained stable in both cases, which strengthens our confidence in the generalizability of the findings.

## Discussion and Implications

In this study, we argued that the societal and situational context in which grandparenting takes place moderates the association between grandchild care and grandparents’ well-being. Specifically, it examined how the link between supplementary grandchild care and grandparents’ well-being was shaped by the COVID-19 pandemic. We tested expectations from role strain theory and role enhancement theory. Role strain theory posits that grandchild care may be straining in general and may have been particularly stressful during the pandemic, with adverse consequences for grandparents’ well-being. By contrast, role enhancement theory posits that grandchild care fosters social integration, which may have been particularly important for grandparents’ well-being when social distancing measures were in place. Drawing on three waves of panel data from the DEAS 2014, 2017, and 2020, we used multigroup maximum likelihood structural equation modeling, a method well suited for isolating a causal effect of grandchild care on well-being from reverse causation and selection due to unobserved heterogeneity.

Our results provide evidence in support of role enhancement theory. Even prior to the COVID-19 pandemic, grandchild care protected grandparents from feelings of loneliness, but we found no associations between grandchild care and life satisfaction or perceived stress. These patterns held for grandmothers and grandfathers alike. Although previous studies reported ambiguous findings (see review by Danielsbacka et al., 2022), this study supplies additional evidence that supplementary grandchild care can be beneficial for grandparents’ social well-being.

During the pandemic, grandchild care gained importance for grandparents’ well-being, and it did so in gendered ways. First, grandchild care became even more important as a protective factor against loneliness. Whereas the direction of this effect and the effect size were similar for grandmothers and grandfathers, it was only statistically significant for grandfathers. Hence, grandchild care as a means for maintaining social integration in the face of restrictions on social interactions seems to have been particularly important for grandfathers. This pattern aligns with previous studies that found that older men are at greater risk of isolation because they are not as embedded in family and social networks as older women and have fewer longstanding relationships (McLaughlin et al., 2010; Sander et al., 2017; Settersten Jr. et al., 2020).

Second, grandchild care was associated with greater life satisfaction, but only among grandmothers. Even though grandchild care protected both grandmothers and grandfathers from loneliness, this protection from loneliness only affected grandmothers’ cognitive judgments of their quality of life. This indicates that grandmothers and grandfathers differ in the criteria they apply to assess their quality of life (Diener et al., 1985). The finding that grandchild care was more consequential for grandmothers’ than grandfathers’ life satisfaction is in line with previous research showing that interpersonal relationships are generally more important for women’s life satisfaction than for men’s (Joshanloo, 2018). It also fits with the observation that grandchild care is often seen as a right and expectation by grandmothers but is viewed as more voluntary for grandfathers (Stelle et al., 2010). Yet the findings diverge from results by Grundy et al. (2012), who found that grandchild care was associated with greater life satisfaction among grandfathers in Chile.

Overall, the results may indicate that grandchild care enabled grandparents to enjoy social integration in the face of social distancing requirements. Yet, the results could also indicate that the loss of contact with grandchildren and adult children was particularly painful during the COVID-19 pandemic and amplified feelings of loneliness. This interpretation is in line with previous research showing that loss of contact with grandchildren (due to the pandemic, but also due to divorce or migration) adversely affected grandparents’ well-being, especially when it was unexpected and when grandparents lacked control over the situation (Di Gessa et al., 2022; Drew & Silverstein, 2007).

By contrast, we found no evidence that grandparents who took care of their grandchildren experienced increased levels of stress out of fear of contracting the virus, as would have been expected by role strain theory. Of course, some grandparents may have used digital devices to perform grandchild care online and thus did not risk contracting the virus from their grandchildren. Yet, even though we lack information on whether grandchild care was performed online or face-to-face, we believe it unlikely that online child care was performed in large numbers. Hence, we assumed that the nonsignificant relationships between grandchild care and perceived stress would also apply to grandparents who had taken care of their grandchildren physically.

When interpreting these results, we need to keep in mind that we only observed grandparents who either had not contracted the virus or had mild courses of disease, as persons who suffered from more serious COVID-19 infections were unlikely to have participated in the survey. Thus, the study cannot be generalized to grandparents who contracted COVID-19 through contact with their children and grandchildren and became seriously ill. Hence, with regards to the “connectivity paradox” (Smith et al., 2020), we found that physical interaction with grandchildren did protect grandparents against social isolation and disconnectedness, but we cannot estimate to what extent this increased their risk of COVID-19 exposure and subsequent illness. A study by Uccheddu and Rizzi (2023) indeed found that grandchild care increased the risk of contracting COVID-19 in a sample of community-dwelling adults aged 50 years or older living in 27 European countries.

Furthermore, the data were limited as they lacked detailed information on caregiving tasks. Information on the number of grandchildren grandparents took care of, as well as characteristics of grandchildren and their parents, such as their health status and socioeconomic background, was not available, and could therefore not be incorporated in the analysis. These factors could significantly influence the burden of caregiving and the well-being of grandparents. Thus, future research should consider these variables to gain a more comprehensive understanding of how grandchild care shapes grandparents’ well-being.

Nevertheless, our results indicate that the societal context matters in structuring the relationship between grandchild care and well-being, as grandchild care became more important for grandparents’ well-being than it was in prepandemic times. Grandparents may have adapted their social expectations and standards during the pandemic and the few contacts they continued to maintain may have had even greater implications for their well-being than before the pandemic. Previous research has already provided some evidence that normative societal expectations about grandparenting moderate associations between grandchild care and grandparents’ quality of life (Neuberger & Haberkern, 2014), whereas family policy regimes appear to be less relevant (Bordone & Arpino, 2019).

Our findings provide further evidence that the contextual situation in which grandchild care takes place the costs and rewards associated with it, and social norms and the opportunity structure seem to matter more in this regard than broad family policies. This suggests that fostering opportunity structures for intergenerational contacts through targeted, low-level initiatives (e.g., volunteer grandparents or grandparents-for-hire initiatives) could be more effective. Additionally, our findings indicate that these programs should address grandmothers and grandfathers alike, as both genders benefit from being involved with grandchildren.

## Supplementary Material

igae101_suppl_Supplementary_Materials

## Data Availability

This publication is based on data from the German Ageing Survey. Data, materials, and the analytic code used in this study are available to the scientific community from the Research Data Center of the German Centre of Gerontology upon request and free of charge (data: https://doi.org/10.5156/DEAS.1996-2021.M.001, materials: https://doi.org/10.5156/DEAS.1996-2021.D.001). The study reported in this manuscript was not preregistered.
