# Supplementary material for: Grandchild Care and Grandparents’ Well-Being in Context: The Impact of the COVID-19 pandemic
Source: Innov Aging. 2024 Nov 2;8(12):igae101. doi: 10.1093/geroni/igae101 (PMC11630762; doi:10.1093/geroni/igae101)
Supplement: igae101_suppl_Supplementary_Materials [file igae101_suppl_supplementary_materials.docx]

***Innovation in Aging* Supplementary Material: Bünning & Huxhold. Grandchild care and grandparents’ well-being in context: The impact of the covid-19 pandemic.**

Table S1. *Descriptive Statistics (Means, Standard Deviations, Valid Cases) of all Dependent and Independent Variables and Survey Participation Rates by Gender and Year of Observation*

|  | Grandfathers | | | Grandmothers | | |
| --- | --- | --- | --- | --- | --- | --- |
|  | Mean | SD | N | Mean | SD | N |
| Loneliness |  |  |  |  |  |  |
| 2014 | 1.76 | 0.50 | 1386 | 1.72 | 0.53 | 1453 |
| 2017 | 1.73 | 0.47 | 1039 | 1.70 | 0.56 | 1054 |
| 2020 | 1.79 | 0.48 | 811 | 1.72 | 0.53 | 860 |
| Stress |  |  |  |  |  |  |
| 2014 | 2.27 | 0.61 | 1385 | 2.41 | 0.68 | 1454 |
| 2017 | 2.22 | 0.57 | 1036 | 2.37 | 0.64 | 1049 |
| 2020 | 2.25 | 0.61 | 804 | 2.39 | 0.67 | 851 |
| Life Satisfaction |  |  |  |  |  |  |
| 2014 | 3.85 | 0.67 | 1398 | 3.84 | 0.74 | 1466 |
| 2017 | 3.90 | 0.63 | 1041 | 3.88 | 0.70 | 1063 |
| 2020 | 3.91 | 0.66 | 808 | 3.98 | 0.63 | 857 |
| Grandchild care |  |  |  |  |  |  |
| 2017 | 0.37 | 0.48 | 1212 | 0.43 | 0.50 | 1242 |
| 2020 | 0.29 | 0.45 | 981 | 0.39 | 0.49 | 1035 |
| Self-rated health |  |  |  |  |  |  |
| 2017 | 3.48 | 0.79 | 1213 | 3.51 | 0.80 | 1244 |
| 2020 | 3.55 | 0.77 | 981 | 3.60 | 0.80 | 1037 |
| Partner in household |  |  |  |  |  |  |
| 2017 | 0.88 | 0.33 | 1214 | 0.72 | 0.45 | 1244 |
| 2020 | 0.88 | 0.32 | 984 | 0.72 | 0.45 | 1037 |
| Employed |  |  |  |  |  |  |
| 2017 | 0.22 | 0.41 | 1213 | 0.30 | 0.46 | 1243 |
| 2020 | 0.22 | 0.41 | 984 | 0.26 | 0.44 | 1035 |
| ISCED level of education |  |  |  |  |  |  |
| Low | 0.03 | 0.16 | 2254 | 0.10 | 0.30 | 2246 |
| Medium | 0.46 | 0.50 | 2254 | 0.55 | 0.50 | 2246 |
| High | 0.51 | 0.50 | 2254 | 0.35 | 0.48 | 2246 |
| Year of birth | 1947.45 | 8.81 | 2254 | 1950.23 | 8.58 | 2246 |
| Participated in survey |  |  |  |  |  |  |
| 2014 | 0.80 | 0.40 | 2254 | 0.81 | 0.40 | 2246 |
| 2017 | 0.54 | 0.50 | 2254 | 0.55 | 0.50 | 2246 |
| 2020 | 0.44 | 0.50 | 2254 | 0.46 | 0.50 | 2246 |
| Valid self-administered questionnaire among those who participated |  |  |  |  |  |  |
| 2014 | 0.78 | 0.41 | 1810 | 0.82 | 0.38 | 1809 |
| 2017 | 0.86 | 0.34 | 1214 | 0.86 | 0.35 | 1244 |
| 2020 | 0.83 | 0.38 | 984 | 0.83 | 0.37 | 1037 |

*Note*: Stress: five-point scale of four items, higher values indicated more elevated stress. Loneliness: four-point scale of six items, higher values indicated greater loneliness. Life satisfaction: five-point scale of five items, higher values indicated greater life satisfaction. Grandchild care: dummy variable (ref: no grandchild care). Partner in household: dummy variable (ref: no partner living in same household). Employed: dummy variable (ref: not employed, retired). Self-rated health: five-point scale, higher values indicate better health. Education: three categories (low, medium, high according to the International Standard Classification of Education (ISCED)).Year of birth: linear (range 1923-1974), divided by ten.

Table S2. *Full Output of Multi-Group Maximum-Likelihood Structural Equation Models: β-Coefficients, Standard Errors and Goodness of Fit Measures by Dependent Variable*

|  |  | Loneliness^a^ | | Stress^b^ | | Life Satisfaction^c^ | |
| --- | --- | --- | --- | --- | --- | --- | --- |
|  |  | β | s.e. | β | s.e. | β | s.e. |
| **Grandfathers** |  |  |  |  |  |  |  |
|  |  |  |  |  |  |  |  |
| Alpha by |  |  |  |  |  |  |  |
| Dependent variable 2017 |  | 1000.00 | 0.00 | 1000.00 | 0.00 | 1000.00 | 0.00 |
| Dependent variable 2020 |  | 1000.00 | 0.00 | 1000.00 | 0.00 | 1000.00 | 0.00 |
|  |  |  |  |  |  |  |  |
| Dependent variable 2017 on |  |  |  |  |  |  |  |
| Dependent variable 2014 |  | 0.13 | 0.10 | 0.11* | 0.05 | 0.15* | 0.15 |
| Child care 2017 |  | -0.10* | 0.04 | 0.02 | 0.05 | 0.08 | 0.08 |
| Partnered 2017 |  | -0.10 | 0.09 | 0.05 | 0.12 | 0.14 | 0.14 |
| Employed 2017 |  | -0.02 | 0.05 | 0.15* | 0.07 | -0.01 | 0.01 |
| Health 2017 |  | 0.00 | 0.02 | -0.08** | 0.03 | 0.09** | 0.09 |
| Education |  | -0.01 | 0.02 | -0.07** | 0.02 | 0.04 | 0.04 |
| Year of birth |  | 0.00 | 0.02 | -0.08*** | 0.02 | 0.03 | 0.03 |
|  |  |  |  |  |  |  |  |
| Dependent variable 2020 on |  |  |  |  |  |  |  |
| Dependent variable 2017 |  | 0.13 | 0.10 | 0.11* | 0.05 | 0.15* | 0.07 |
| Child care 2020 |  | -0.26** | 0.09 | 0.02 | 0.05 | 0.08 | 0.05 |
| Partnered 2020 |  | -0.10 | 0.09 | 0.05 | 0.12 | 0.14 | 0.12 |
| Employed 2020 |  | -0.02 | 0.05 | 0.15* | 0.07 | -0.01 | 0.07 |
| Health 2020 |  | 0.00 | 0.02 | -0.08** | 0.03 | 0.09** | 0.03 |
| Education |  | -0.01 | 0.02 | -0.07** | 0.02 | 0.04 | 0.02 |
| Year of birth |  | 0.00 | 0.02 | -0.08*** | 0.02 | 0.03 | 0.02 |
|  |  |  |  |  |  |  |  |
| Education on |  |  |  |  |  |  |  |
| Dependent variable 2014 |  | -0.09** | 0.03 | -0.13*** | 0.03 | 0.07** | 0.02 |
| Child care 2017 |  | 0.02 | 0.04 | 0.01 | 0.04 | 0.01 | 0.04 |
| Child care 2020 |  | -0.10* | 0.04 | -0.09* | 0.04 | -0.09* | 0.04 |
| Partnered 2017 |  | -0.04 | 0.11 | -0.04 | 0.11 | -0.07 | 0.11 |
| Partnered 2020 |  | 0.03 | 0.11 | 0.03 | 0.11 | 0.03 | 0.11 |
| Employed 2017 |  | -0.07 | 0.06 | -0.07 | 0.06 | -0.07 | 0.06 |
| Employed 2020 |  | 0.01 | 0.07 | 0.01 | 0.07 | 0.00 | 0.07 |
| Health 2017 |  | 0.07** | 0.03 | 0.06* | 0.03 | 0.06* | 0.03 |
| Health 2020 |  | 0.04 | 0.03 | 0.02 | 0.03 | 0.03 | 0.03 |
|  |  |  |  |  |  |  |  |
| Year of birth on |  |  |  |  |  |  |  |
| Dependent variable 2014 |  | 0.07 | 0.04 | -0.01 | 0.03 | -0.16 *** | 0.03 |
| Child care 2017 |  | 0.12** | 0.04 | 0.12** | 0.04 | 0.12** | 0.04 |
| Child care 2020 |  | 0.17** | 0.05 | 0.16** | 0.05 | 0.17*** | 0.05 |
| Partnered 2017 |  | -0.26* | 0.12 | -0.27* | 0.12 | -0.21 | 0.12 |
| Partnered 2020 |  | 0.30* | 0.12 | 0.31* | 0.12 | 0.30* | 0.12 |
| Employed 2017 |  | 0.93*** | 0.06 | 0.93*** | 0.06 | 0.91*** | 0.06 |
| Employed 2020 |  | 0.70*** | 0.07 | 0.70*** | 0.07 | 0.70*** | 0.07 |
| Health 2017 |  | -0.04 | 0.03 | -0.04 | 0.03 | -0.01 | 0.03 |
| Health 2020 |  | 0.02 | 0.03 | 0.02 | 0.03 | 0.03 | 0.03 |
|  |  |  |  |  |  |  |  |
| Alpha with |  |  |  |  |  |  |  |
| Dependent variable 2014 |  | 0.13*** | 0.02 | 0.16*** | 0.02 | 0.22*** | 0.03 |
| Child care 2017 |  | 0.02 | 0.01 | 0.00 | 0.01 | -0.01 | 0.01 |
| Child care 2020 |  | 0.05* | 0.02 | 0.00 | 0.01 | 0.00 | 0.01 |
| Partnered 2017 |  | 0.00 | 0.01 | -0.01 | 0.01 | 0.01 | 0.01 |
| Partnered 2020 |  | 0.00 | 0.01 | -0.01 | 0.01 | 0.01 | 0.01 |
| Employed 2017 |  | 0.00 | 0.01 | -0.02 | 0.02 | 0.00 | 0.02 |
| Employed 2020 |  | -0.01 | 0.01 | -0.03* | 0.01 | 0.00 | 0.01 |
| Health 2017 |  | -0.07 | 0.02*** | -0.09*** | 0.02 | 0.11*** | 0.02 |
| Health 2020 |  | -0.05 | 0.02** | -0.09*** | 0.02 | 0.09*** | 0.02 |
| Education |  | 0.00 | 0.00 | 0.00 | 0.00 | 0.00 | 0.00 |
| Year of birth |  | 0.00 | 0.00 | 0.00 | 0.00 | 0.00 | 0.00 |
|  |  |  |  |  |  |  |  |
| Child care 2020 with |  |  |  |  |  |  |  |
| Dependent variable 2017 |  | -0.05** | 0.02 | -0.01 | 0.01 | 0.00 | 0.01 |
| Dependent variable 2014 |  | -0.02 | 0.01 | 0.00 | 0.01 | 0.02 | 0.01 |
| Child care 2017 |  | 0.08*** | 0.01 | 0.08*** | 0.01 | 0.08*** | 0.01 |
|  |  |  |  |  |  |  |  |
| Child care 2017 with |  |  |  |  |  |  |  |
| Dependent variable 2014 |  | -0.01 | 0.01 | -0.01 | 0.01 | 0.02 | 0.01 |
|  |  |  |  |  |  |  |  |
| Education with |  |  |  |  |  |  |  |
| Year of birth |  | -0.03*** | 0.01 | -0.03*** | 0.01 | -0.03** | 0.01 |
|  |  |  |  |  |  |  |  |
| Partnered 2017 with |  |  |  |  |  |  |  |
| Dependent variable 2014 |  | -0.01* | 0.01 | -0.01 | 0.01 | 0.04*** | 0.01 |
| Child care 2017 |  | 0.02*** | 0.00 | 0.02*** | 0.00 | 0.02*** | 0.00 |
| Child care 2020 |  | 0.01* | 0.01 | 0.01 | 0.01 | 0.01 | 0.01 |
|  |  |  |  |  |  |  |  |
| Partnered 2020 with |  |  |  |  |  |  |  |
| Dependent variable 2014 |  | -0.01 | 0.01 | -0.01 | 0.01 | 0.04*** | 0.01 |
| Child care 2017 |  | 0.02*** | 0.01 | 0.02*** | 0.01 | 0.02*** | 0.01 |
| Child care 2020 |  | 0.01 | 0.01 | 0.01 | 0.01 | 0.01 | 0.01 |
| Partnered 2017 |  | 0.09*** | 0.00 | 0.09*** | 0.00 | 0.09*** | 0.00 |
|  |  |  |  |  |  |  |  |
| Employed 2017 with |  |  |  |  |  |  |  |
| Dependent variable 2014 |  | 0.00 | 0.01 | 0.02 | 0.01 | -0.01 | 0.01 |
| Child care 2017 |  | -0.01* | 0.01 | -0.01* | 0.01 | -0.01* | 0.01 |
| Child care 2020 |  | 0.01 | 0.01 | 0.01 | 0.01 | 0.01 | 0.01 |
| Partnered 2017 |  | 0.01 | 0.00 | 0.01 | 0.00 | 0.01 | 0.00 |
| Partnered 2020 |  | 0.01* | 0.00 | 0.01** | 0.00 | 0.01* | 0.00 |
|  |  |  |  |  |  |  |  |
| Employed 2020 with |  |  |  |  |  |  |  |
| Dependent variable 2014 |  | 0.00 | 0.01 | 0.01 | 0.01 | 0.01 | 0.01 |
| Child care 2017 |  | -0.02* | 0.01 | -0.02* | 0.01 | -0.02* | 0.01 |
| Child care 2020 |  | 0.00 | 0.01 | 0.00 | 0.01 | 0.00 | 0.01 |
| Partnered 2017 |  | 0.00 | 0.00 | 0.00 | 0.00 | 0.00 | 0.00 |
| Partnered 2020 |  | 0.01 | 0.00 | 0.01 | 0.00 | 0.01 | 0.00 |
| Employed 2017 |  | 0.13*** | 0.01 | 0.13*** | 0.01 | 0.13*** | 0.01 |
|  |  |  |  |  |  |  |  |
| Health 2017 with |  |  |  |  |  |  |  |
| Dependent variable 2014 |  | -0.07*** | 0.01 | -0.14*** | 0.02 | 0.17*** | 0.02 |
| Child care 2017 |  | 0.01 | 0.01 | 0.01 | 0.01 | 0.01 | 0.01 |
| Child care 2020 |  | 0.01 | 0.01 | 0.00 | 0.01 | 0.00 | 0.01 |
| Partnered 2017 |  | 0.01 | 0.01 | 0.01 | 0.01 | 0.01 | 0.01 |
| Partnered 2020 |  | 0.00 | 0.01 | 0.01 | 0.01 | 0.00 | 0.01 |
| Employed 2017 |  | 0.02* | 0.01 | 0.03** | 0.01 | 0.03* | 0.01 |
| Employed 2020 |  | 0.04** | 0.01 | 0.04** | 0.01 | 0.04** | 0.01 |
|  |  |  |  |  |  |  |  |
| Health 2020 with |  |  |  |  |  |  |  |
| Dependent variable 2014 |  | -0.04* | 0.02 | -0.11*** | 0.02 | 0.12*** | 0.02 |
| Child care 2017 |  | -0.02 | 0.01 | -0.02 | 0.01 | -0.02 | 0.01 |
| Child care 2020 |  | 0.03* | 0.01 | 0.02* | 0.01 | 0.02 | 0.01 |
| Partnered 2017 |  | 0.01 | 0.01 | 0.01 | 0.01 | 0.01 | 0.01 |
| Partnered 2020 |  | 0.01 | 0.01 | 0.01 | 0.01 | 0.01 | 0.01 |
| Employed 2017 |  | 0.04*** | 0.01 | 0.04*** | 0.01 | 0.04** | 0.01 |
| Employed 2020 |  | 0.04*** | 0.01 | 0.04*** | 0.01 | 0.04*** | 0.01 |
| Health 2017 |  | 0.34*** | 0.02 | 0.34*** | 0.02 | 0.34*** | 0.02 |
|  |  |  |  |  |  |  |  |
| **Grandmothers** |  |  |  |  |  |  |  |
|  |  |  |  |  |  |  |  |
| Alpha by |  |  |  |  |  |  |  |
| Dependent variable 2017 |  | 1000.00 | 0.00 | 1000.00 | 0.00 | 1000.00 | 0.00 |
| Dependent variable 2020 |  | 1000.00 | 0.00 | 1000.00 | 0.00 | 1000.00 | 0.00 |
|  |  |  |  |  |  |  |  |
| Dependent variable 2017 on |  |  |  |  |  |  |  |
| Dependent variable 2014 |  | 0.51*** | 0.14 | 0.18*** | 0.05 | 0.14* | 0.06 |
| Child care 2017 |  | -0.10* | 0.04 | 0.02 | 0.05 | 0.08 | 0.05 |
| Partnered 2017 |  | 0.12 | 0.09 | 0.14 | 0.11 | 0.06 | 0.10 |
| Employed 2017 |  | 0.12 | 0.06 | 0.17* | 0.07 | 0.11 | 0.07 |
| Health 2017 |  | -0.05 | 0.03 | -0.10** | 0.04 | 0.05 | 0.03 |
| Education |  | -0.01 | 0.02 | -0.07** | 0.02 | 0.01 | 0.02 |
| Year of birth |  | 0.02 | 0.02 | -0.02 | 0.03 | 0.01 | 0.03 |
|  |  |  |  |  |  |  |  |
| Dependent variable 2020 on |  |  |  |  |  |  |  |
| Dependent variable 2017 |  | 0.51*** | 0.14 | 0.18*** | 0.05 | 0.14* | 0.06 |
| Child care 2020 |  | -0.26** | 0.09 | 0.02 | 0.05 | 0.35*** | 0.09 |
| Partnered 2020 |  | 0.12 | 0.09 | 0.14 | 0.11 | 0.06 | 0.10 |
| Employed 2020 |  | 0.12 | 0.06 | 0.17* | 0.07 | 0.11 | 0.07 |
| Health 2020 |  | -0.05 | 0.03 | -0.10** | 0.04 | 0.05 | 0.03 |
| Education |  | -0.01 | 0.02 | -0.07** | 0.02 | 0.01 | 0.02 |
| Year of birth |  | 0.02 | 0.02 | -0.02 | 0.03 | 0.01 | 0.03 |
|  |  |  |  |  |  |  |  |
| Education on |  |  |  |  |  |  |  |
| Dependent variable 2014 |  | -0.11*** | 0.03 | -0.10*** | 0.03 | 0.05 | 0.03 |
| Child care 2017 |  | -0.06 | 0.04 | -0.05 | 0.04 | -0.05 | 0.04 |
| Child care 2020 |  | 0.09 | 0.05 | 0.09* | 0.05 | 0.09* | 0.05 |
| Partnered 2017 |  | -0.04 | 0.11 | -0.01 | 0.11 | -0.02 | 0.11 |
| Partnered 2020 |  | 0.13 | 0.11 | 0.09 | 0.11 | 0.11 | 0.11 |
| Employed 2017 |  | 0.05 | 0.06 | 0.05 | 0.06 | 0.05 | 0.06 |
| Employed 2020 |  | 0.05 | 0.07 | 0.06 | 0.07 | 0.05 | 0.07 |
| Health 2017 |  | 0.05 | 0.03 | 0.04 | 0.03 | 0.05 | 0.03 |
| Health 2020 |  | 0.04 | 0.03 | 0.04 | 0.03 | 0.04 | 0.03 |
|  |  |  |  |  |  |  |  |
| Year of birth on |  |  |  |  |  |  |  |
| Dependent variable 2014 |  | 0.11** | 0.03 | 0.05 | 0.03 | -0.11*** | 0.03 |
| Child care 2017 |  | 0.05 | 0.04 | 0.04 | 0.04 | 0.04 | 0.04 |
| Child care 2020 |  | 0.17*** | 0.05 | 0.16*** | 0.05 | 0.16** | 0.05 |
| Partnered 2017 |  | -0.12 | 0.11 | -0.15 | 0.11 | -0.10 | 0.10 |
| Partnered 2020 |  | 0.32** | 0.11 | 0.34** | 0.11 | 0.32** | 0.11 |
| Employed 2017 |  | 0.72*** | 0.06 | 0.73*** | 0.06 | 0.73*** | 0.06 |
| Employed 2020 |  | 0.66*** | 0.06 | 0.65*** | 0.06 | 0.64*** | 0.06 |
| Health 2017 |  | -0.07* | 0.03 | -0.07* | 0.03 | -0.06* | 0.03 |
| Health 2020 |  | 0.00 | 0.03 | -0.01 | 0.03 | 0.01 | 0.03 |
|  |  |  |  |  |  |  |  |
| Alpha with |  |  |  |  |  |  |  |
| Dependent variable 2014 |  | 0.06 | 0.04 | 0.15*** | 0.02 | 0.26*** | 0.03 |
| Child care 2017 |  | 0.01 | 0.01 | -0.01 | 0.01 | -0.02 | 0.01 |
| Child care 2020 |  | 0.04 | 0.02 | -0.03 | 0.02 | -0.09*** | 0.02 |
| Partnered 2017 |  | -0.05* | 0.02 | -0.04 | 0.02 | 0.03 | 0.02 |
| Partnered 2020 |  | -0.05* | 0.02 | -0.05* | 0.02 | 0.03 | 0.02 |
| Employed 2017 |  | -0.04* | 0.02 | -0.04* | 0.02 | -0.02 | 0.02 |
| Employed 2020 |  | -0.03* | 0.01 | -0.03 | 0.02 | -0.03 | 0.02 |
| Health 2017 |  | -0.03 | 0.02 | -0.11*** | 0.03 | 0.12*** | 0.03 |
| Health 2020 |  | -0.02 | 0.03 | -0.11*** | 0.03 | 0.11*** | 0.03 |
| Education |  | 0.00 | 0.00 | 0.00 | 0.00 | 0.00 | 0.00 |
| Year of birth |  | 0.00 | 0.00 | 0.00 | 0.00 | 0.00 | 0.00 |
|  |  |  |  |  |  |  |  |
| Child care 2020 with |  |  |  |  |  |  |  |
| Dependent variable 2017 |  | -0.05* | 0.02 | 0.00 | 0.02 | 0.08*** | 0.02 |
| Dependent variable 2014 |  | -0.02 | 0.01 | -0.01 | 0.01 | 0.00 | 0.01 |
| Child care 2017 |  | 0.10*** | 0.01 | 0.10*** | 0.01 | 0.10*** | 0.01 |
|  |  |  |  |  |  |  |  |
| Child care 2017 with |  |  |  |  |  |  |  |
| Dependent variable 2014 |  | -0.02** | 0.01 | -0.01 | 0.01 | 0.01 | 0.01 |
|  |  |  |  |  |  |  |  |
| Education with |  |  |  |  |  |  |  |
| Year of birth |  | 0.01 | 0.01 | 0.01 | 0.01 | 0.01 | 0.01 |
|  |  |  |  |  |  |  |  |
| Partnered 2017 with |  |  |  |  |  |  |  |
| Dependent variable 2014 |  | -0.01 | 0.01 | -0.01 | 0.01 | 0.06*** | 0.01 |
| Child care 2017 |  | 0.02** | 0.01 | 0.02** | 0.01 | 0.02** | 0.01 |
| Child care 2020 |  | 0.02** | 0.01 | 0.02** | 0.01 | 0.02* | 0.01 |
|  |  |  |  |  |  |  |  |
| Partnered 2020 with |  |  |  |  |  |  |  |
| Dependent variable 2014 |  | -0.01 | 0.01 | -0.02* | 0.01 | 0.05*** | 0.01 |
| Child care 2017 |  | 0.03*** | 0.01 | 0.03*** | 0.01 | 0.03*** | 0.01 |
| Child care 2020 |  | 0.03*** | 0.01 | 0.03*** | 0.01 | 0.02** | 0.01 |
| Partnered 2017 |  | 0.18*** | 0.01 | 0.18*** | 0.01 | 0.19*** | 0.01 |
|  |  |  |  |  |  |  |  |
| Employed 2017 with |  |  |  |  |  |  |  |
| Dependent variable 2014 |  | 0.01 | 0.01 | 0.01 | 0.01 | -0.01 | 0.01 |
| Child care 2017 |  | 0.02* | 0.01 | 0.02* | 0.01 | 0.02* | 0.01 |
| Child care 2020 |  | 0.02** | 0.01 | 0.02* | 0.01 | 0.02* | 0.01 |
| Partnered 2017 |  | 0.02*** | 0.01 | 0.02*** | 0.01 | 0.02*** | 0.01 |
| Partnered 2020 |  | 0.03*** | 0.01 | 0.04*** | 0.01 | 0.04*** | 0.01 |
|  |  |  |  |  |  |  |  |
| Employed 2020 with |  |  |  |  |  |  |  |
| Dependent variable 2014 |  | 0.00 | 0.01 | 0.01 | 0.01 | -0.01 | 0.01 |
| Child care 2017 |  | 0.02** | 0.01 | 0.02** | 0.01 | 0.02** | 0.01 |
| Child care 2020 |  | 0.02** | 0.01 | 0.02** | 0.01 | 0.02** | 0.01 |
| Partnered 2017 |  | 0.02* | 0.01 | 0.02* | 0.01 | 0.02* | 0.01 |
| Partnered 2020 |  | 0.02** | 0.01 | 0.02** | 0.01 | 0.02** | 0.01 |
| Employed 2017 |  | 0.16*** | 0.01 | 0.16*** | 0.01 | 0.16*** | 0.01 |
|  |  |  |  |  |  |  |  |
| Health 2017 with |  |  |  |  |  |  |  |
| Dependent variable 2014 |  | -0.07*** | 0.01 | -0.17*** | 0.02 | 0.18*** | 0.02 |
| Child care 2017 |  | 0.05*** | 0.01 | 0.05*** | 0.01 | 0.05*** | 0.01 |
| Child care 2020 |  | 0.02 | 0.01 | 0.03 | 0.01 | 0.02 | 0.01 |
| Partnered 2017 |  | 0.03** | 0.01 | 0.03** | 0.01 | 0.03** | 0.01 |
| Partnered 2020 |  | 0.04** | 0.01 | 0.04** | 0.01 | 0.04** | 0.01 |
| Employed 2017 |  | 0.05*** | 0.01 | 0.05*** | 0.01 | 0.05*** | 0.01 |
| Employed 2020 |  | 0.06*** | 0.01 | 0.07*** | 0.01 | 0.06*** | 0.01 |
|  |  |  |  |  |  |  |  |
| Health 2020 with |  |  |  |  |  |  |  |
| Dependent variable 2014 |  | -0.09*** | 0.02 | -0.15*** | 0.02 | 0.20*** | 0.02 |
| Child care 2017 |  | 0.05** | 0.01 | 0.05*** | 0.01 | 0.05*** | 0.01 |
| Child care 2020 |  | 0.03* | 0.01 | 0.03** | 0.01 | 0.03* | 0.01 |
| Partnered 2017 |  | 0.02 | 0.01 | 0.02 | 0.01 | 0.02 | 0.01 |
| Partnered 2020 |  | 0.03** | 0.01 | 0.03** | 0.01 | 0.03** | 0.01 |
| Employed 2017 |  | 0.05*** | 0.01 | 0.05*** | 0.01 | 0.05*** | 0.01 |
| Employed 2020 |  | 0.05*** | 0.01 | 0.05*** | 0.01 | 0.05*** | 0.01 |
| Health 2017 |  | 0.38*** | 0.02 | 0.39*** | 0.02 | 0.39*** | 0.02 |
|  |  |  |  |  |  |  |  |
| Goodness of Fit Measures |  |  |  |  |  |  |  |
| RMSEA |  | 0.005 |  | 0.024 |  | 0.025 |  |
| CFI |  | 1.000 |  | 0.994 |  | 0.995 |  |

*Note:* Stress: five-point scale of four items, higher values indicated more elevated stress. Loneliness: four-point scale of six items, higher values indicated greater loneliness. Life satisfaction: five-point scale of five items, higher values indicated greater life satisfaction. Child care: dummy variable (ref: no child care). Partnered: dummy variable (ref: not living with a partner in the same household). Employed: dummy variable (ref: not employed, retired). Health: five-point scale of self-rated health, higher values indicate better health. Education: three categories (low, medium, high according to the International Standard Classification of Education (ISCED)). Year of birth: linear (range 1923-1974), divided by ten. Alpha: latent variable, which had unrestricted correlations with all time-varying predictors (person-specific fixed effect). All control variables were centered around their grand mean in 2017 and their effects were allowed to vary between men and women.

^a^ All child care coefficients (β_Childcare 2017 Men,_ β_Childcare 2017 Women,_ β_Childcare 2020 Men,_ and β_Childcare 2020 Women_) set equal.
^b^ β_Childcare 2017 Men_ and β_Childcare 2017 Women_ set equal_,_ β_Childcare 2020 Men,_ and β_Childcare 2020 Women_ set equal.
^c^ β_Childcare 2017 Men_, β_Childcare 2020 Men_, and β_Childcare 2020 Women_ set equal.
* *p* < .05, ** *p* < .01, *** *p* < .001.
